# Amalgamation of Geometry Structure, Meteorological and Thermophysical Parameters for Intelligent Prediction of Temperature Fields in 3D Scenes

**DOI:** 10.3390/s22062386

**Published:** 2022-03-20

**Authors:** Yuan Cao, Ligang Li, Wei Ni, Bo Liu, Wenbo Zhou, Qi Xiao

**Affiliations:** 1 National Space Science Center, Key Laboratory of Electronics and Information Technology for Space System, Chinese Academy of Sciences, Beijing 100190, China; caoyuan20@mails.ucas.ac.cn (Y.C.); niwei@nssc.ac.cn (W.N.); liubo183@mails.ucas.ac.cn (B.L.); zhouwenbo20@mails.ucas.ac.cn (W.Z.); xiaoqi19@mails.ucas.ac.cn (Q.X.); 2 School of Computer Science and Technology, University of Chinese Academy of Sciences, Beijing 100049, China

**Keywords:** three-dimensional scene, temperature field, intelligent prediction, network, geometry structure, meteorological parameters, thermophysical parameters

## Abstract

Temperature field calculation is an important step in infrared image simulation. However, the existing solutions, such as heat conduction modelling and pre-generated lookup tables based on temperature calculation tools, are difficult to meet the requirements of high-performance simulation of infrared images based on three-dimensional scenes under multi-environmental conditions in terms of accuracy, timeliness, and flexibility. In recent years, machine learning-based temperature field prediction methods have been proposed, but these methods only consider the influence of meteorological parameters on the temperature value, while not considering the geometric structure and the thermophysical parameters of the object, which results in the low accuracy. In this paper, a multivariate temperature field prediction network based on heterogeneous data (MTPHNet) is proposed. The network fuses geometry structure, meteorological, and thermophysical parameters to predict temperature. First, a Point Cloud Feature Extraction Module and Environmental Data Mapping Module are used to extract geometric information, thermophysical, and meteorological features. The extracted features are fused by the Data Fusion Module for temperature field prediction. Experiment results show that MTPHNet significantly improves the prediction accuracy of the temperature field. Compared with the v-Support Vector Regression and the combined back-propagation neural network, the mean absolute error and root mean square error of MTPHNet are reduced by at least 23.4% and 27.7%, respectively, while the R-square is increased by at least 5.85%. MTPHNet also achieves good results in multi-target and complex target temperature field prediction tasks. These results validate the effectiveness of the proposed method.

## 1. Introduction

Infrared imaging technology has the characteristics of high penetration, strong anti-interference, good concealment, and high precision, which can significantly compensate for visible-light imaging technology’s lack of night vision capability. With the rapid development of infrared imaging technologies, infrared imaging systems have been widely applied to military, industrial, and civilian applications [1]. To develop such systems, it is essential to obtain the appropriate system parameters in advance. This requires a large number of sample images under different lighting conditions for testing and evaluation. However, owing to the complex influences of region, scenery, time-of-day and meteorological conditions, obtaining a sufficient number of samples often requires extensive re-sources and labor. Under extreme conditions, it is impossible to obtain a sufficient number of test samples. To overcome this limitation, infrared simulation has been proposed. It obtains infrared images by simulating actual infrared imaging processes by traversing three-dimensional (3D) scene construction, temperature field and radiation calculations, atmospheric radiation transmission calculation, and imaging instrument simulation. Among these, the temperature field calculation is the most important.

Research on temperature field calculations has undergone a remarkable evolution. Initially, researchers used empirical or semi-empirical models to calculate a temperature field. For instance, Jacobs [2] used a one-dimensional thermal model to calculate the temperatures of simple geometries. Biesel and Rohlfing [3] obtained an object’s surface temperature by setting a series of assumptions for the heat balance equation. Curtis and Rive-ra [4] established an empirical surface temperature model that comprehensively considers the influences of time, material type, meteorological conditions, and object orientation. Balfour and Bushlin [5] established a general expression of surface temperature with respect to the sun, sky, air temperature and wind speed. However, these models are labor- and resource-intensive. Moreover, they cannot adapt to changes in details, and their accuracy is low.

To meet the requirements of accuracy, first-principle models were used for temperature field calculations. This model is based on the principle of heat transfer. The heat balance equation is established by considering various factors that affect the temperature change of the object; the temperature value is calculated by numerical calculations. For instance, Gonda et al. [6] introduced the temperature prediction model, which uses a hot node network method to calculate the temperature field distribution on the surface of an object. Sheffer and Cathcart [7] developed a thermal calculation model using a first-principle model, which considers factors, such as solar and sky radiation, mass transfer process, fluid transmission, occlusion, and multiple reflections, and can more accurately obtain the temperature change of the object. Currently, several commercial temperature field calculation software programs, such as TAITherm (https://thermoanalytics.com/taitherm, accessed on 27 February 2022) [8], Fluent (https://www.ansys.com/zh-cn/products/fluids/ansys-fluent, accessed on 27 February 2022), and Vega, which are based on first-principle models, have been developed. They realize high-precision target temperature field calculations by setting thermophysical and meteorological parameters. However, for calculating a temperature field to deter-mine a target, it is usually necessary to input several parameters, such as material, thickness, shape, atmospheric temperature, and wind speed and direction. This impedes calculations at different periods and under varying meteorological conditions, and over-whelms the current GPUs. Hence, it cannot support real-time infrared simulations.

Considering the first-principle models’ calculation speed bottleneck, Hu et al. [9] proposed a scheme that uses the temperature field calculation method to generate the temperature data of a typical target scene under typical environmental conditions in advance and save it in a database lookup table. The temperature value is then obtained using database interpolation. A look-up table significantly increases the simulation speed, but it is limited in its ability to accommodate sampling resolution design and interpolation methods with numerous input meteorological parameters. Moreover, accuracy cannot be guaranteed if only the main input parameters are considered.

The temperature field calculation method proposed in this study is based on machine learning and is designed to meet real-time, high-precision and flexible infrared simulation requirements. It uses a data-driven approach to establish a mapping of parameters affecting the model’s temperature field distribution to the model temperature, which essentially fits the heat-balance equation established by the first-principle model. Huang and Wu [10] proposed a similar method based on a combined back-propagation (BP) neural network to establish a relationship between model temperature and meteorological parameters. Huang et al. [11] screened meteorological parameters using the heat balance equation and used the ν-support vector regression (v-SVR) model [12] to fit the model temperature. This meets the real-time requirements of simulations. However, contemporary machine learning models only consider the influence of meteorological parameters on temperature and ignore the influence of other factors, which affects accuracy.

This study provides a novel temperature field calculation method based on machine learning for high-precision real-time prediction of temperature field under the influence of multiple environmental variables in the real-time simulation of a 3D scene’s infrared im-aging. It addresses the limitations of the contemporary models by comprehensively considering geometry structure, meteorological, and thermophysical parameters, which meets the requirements of real-time and accurate temperature field prediction. The main contributions of this study are as follows:

(1) A multivariate temperature field prediction network based on heterogeneous data (MTPHNet), which combines the characteristics of heterogeneous thermo-physical and meteorological data as 3D model parameters to predict temperature using fusion features and to improve model generalizability;
(2) To solve the problem of memory explosion when the Transformer (http://nlp.seas.harvard.edu/2018/04/03/attention.html, accessed on 27 February 2022) structure deals with 3D model thermophysical parameters, we propose the PointNet (https://github.com/charlesq34/pointnet, accessed on 27 February 2022) structure as the 3D model thermophysical feature extraction module and imitate the parameter sharing idea of a convolutional neural network to extract local and global features separately. The final fitting effect proves the effectiveness of the method;
(3) We used a multilayer perceptron (MLP) module to map the meteorological parameters to fuse the meteorological and thermophysical parameters so that the mapped features and thermophysical parameters have the same size, which is convenient for the subsequent fusion process.

The experimental results validate the effectiveness of our proposed algorithm. The remainder of this article is organized as follows: Section 2 describes our analysis process and the proposed method in detail. In Section 3, the data formats, evaluation metrics, and training methods used for training are introduced. In Section 4, corresponding experiments are designed to verify the effectiveness of this method, and the experimental results are analyzed and discussed. Section 5 draws some conclusions about our method.

## 2. Materials and Methods

### 2.1. Analysis of the Parameters That Affect the Temperature Field Distribution of the 3D Model in the Natural Environment

#### 2.1.1. Calculation Principle

A series of heat transfer processes with different mechanisms occur between the surface of a 3D model in a natural environment and the atmospheric boundary layer. A 3D model comprises different materials; the methods and speeds of heat exchange between different materials and the external environment are different.

**Figure 1:**
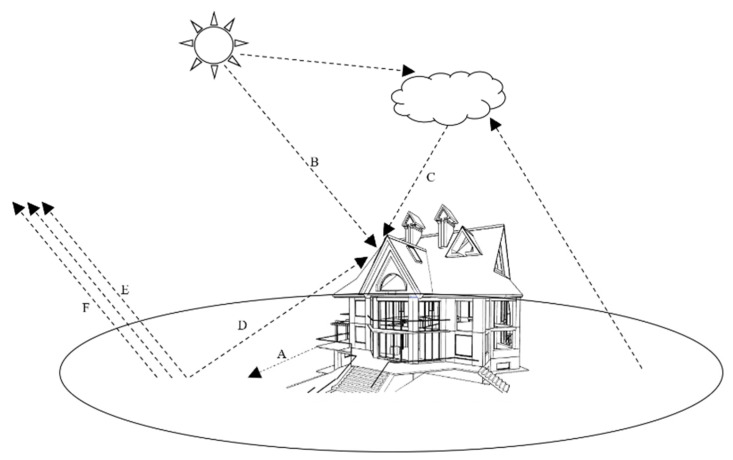
**A** 3D model of processes of energy interactions to reach thermal equilibrium under natural conditions: (A) heat transferred by temperature differences between objects; (B) energy directly transmitted to objects by solar radiation; (C) energy transferred by particles to objects after absorbing external radiation; (D) heat radiation energy transferred from surrounding objects to the target object; (E) energy transferred by fluid flow; and (F) energy transferred by water evaporation, water vapor condensation and migration.

Figure 1 illustrates the energy interactions between an object and the external environment, which ultimately results in thermal equilibrium. For example, temperature differences between objects cause heat transfer (Figure 1A). Energy can also be transmitted directly to objects by solar radiation (Figure 1B). Atmospheric particles can also transfer energy to objects after absorbing external radiation (Figure 1C). Heat energy can be transferred from surrounding objects to the target object (Figure 1D). Fluid flow also contributes to energy transfer (Figure 1E). Lastly, energy transfer can be caused by the evaporation of water, water vapor condensation, and migration (Figure 1F).

Based on the law of the conservation of energy and the processes illustrated, the heat balance equation of an object’s surface is as follows:

(1) $${k_{i}\frac{\partial T}{\partial n}|}_{i}=a_{s}E_{sun}+a_{l}E_{sky}+\overset{M}{\underset{j=1}{\sum }}Q_{rj}-\epsilon \sigma T^{4}\pm Q_{c}\pm Q_{ec}$$

where $k_{i}\frac{\partial T}{\partial n}|$ is the heat conduction of the object, $a_{s}E_{sun}$ denotes the ability of an object to absorb solar radiation, $a_{l}E_{sky}$ denotes the ability of an object to absorb radiation from the sky, $\sum_{j=1}^{M}Q_{rj}$ denotes the radiative heat transfer from other objects around, $\epsilon \sigma T^{4}$ denotes the self-radiation of the object, $Q_{c}$ denotes convective heat transfer, and $Q_{ec}$ denotes hidden heat.

With Equation (1), when the boundary conditions at each moment are known, the temperature field distribution at each moment can be calculated. The calculation result at the current moment is also the boundary condition at the next moment. By analyzing the above-mentioned energy transfer process, we can filter the variables that play a key role in the calculation of the temperature field distribution.

By analyzing the above-mentioned energy transfer process, we can filter out the variables that play a key role in the calculation of the temperature field distribution:

(A) Heat conduction: Owing to the collision of numerous molecules and subatomic particles, energy flows from a high-temperature object to a low-temperature object. For a temperature change caused by heat conduction, the main influencing factors are the properties of the object itself, including thermal conductivity, thickness, shape, etc.

(B) Sun radiation: Objects absorb radiant energy from the sun, which is a form of radiant heat transfer. When the object is on a clear and cloudless level surface, the formula is as follows:

(2) $$E_{s0}=\left[1-A\left(U^{*},\beta \right)\right]\left(0.349E_{0}\right)sin\beta +\left(\frac{1-\rho_{0}}{1-\rho_{0}\bar{\rho_{g}}}\right)\left(0.651E_{0}\right)sin\beta$$

where $E_{0}$ denotes the solar radiation of the entire waveband, $A\left(U^{*},\beta \right)$ denotes the absorbable coefficient, which is a function of relative humidity, air temperature, and solar altitude, β denotes the solar elevation angle, $\bar{\rho_{g}}$ denotes the reflectivity of the ground, and $\rho_{0}$ is the Rayleigh reflectivity of the atmosphere, which is a function of the solar elevation angle.

Considering the cloudy sky, Equation (2) is modified to obtain the following formula:

(3) $$E_{fsun}=E_{s0}\cdot CF$$

where CF is a function related to cloud coverage.

Therefore, the main factors influencing the temperature changes caused by solar radiation are relative humidity, air temperature, solar altitude angle, and cloud coverage. The solar altitude angle is related to the longitude, latitude, time zone, and date. In this study, it is assumed that the temperature field is calculated in a fixed scene; hence, the longitude, latitude, and time zone are invariant. Therefore, the main influencing factors of temperature changes caused by solar radiation are relative humidity, air temperature, date, and cloud coverage.

(C) Sky radiation: Atmospheric particles, such as carbon dioxide and water vapor, are present in the atmosphere. These particles absorb external radiation; thus, they have a certain temperature. Therefore, sky radiation is essentially generated by the thermal radiation of atmospheric particles, and it affects objects on the ground. The formula for sky radiation is as follows:

(4) $$E_{sky}=\left(a+b\sqrt{e}\right)\sigma T_{a}^{4}$$

where $T_{a}$ is the sky temperature, which can be calculated from cloud coverage, atmospheric temperature, humidity, and altitude; a and b are related to the location and time of the measurement, and e is a function of relative humidity and atmospheric temperature.

Because altitude and location are constant in this study, the temperature changes caused by sky radiation are related to cloud coverage, atmospheric temperature, humidity, and time.

(D) Radiation from other objects: When the temperature of an object is higher than absolute zero, it spontaneously radiates energy. Therefore, when there are other objects around it, it is affected by their radiation. Hence, it is necessary to obtain the surrounding objects’ temperature data.

(E) Convection heat transfer: Fluid flow further affects temperature changes. For ground objects, the main influencing factors of temperature changes caused by convective heat transfer are wind speed and direction, and air temperature.

(F) Latent heat is the energy transfer caused by the evaporation of water; and condensation and migration of water vapor. The object studied in this study does not involve heat exchange in this area.

#### 2.1.2. Determination of the Parameters That Affect the Surface Temperature Field of the Object

Because this study focuses on the calculation of a 3D target’s temperature field at a fixed altitude and location, the main meteorological parameters are date, atmospheric temperature, solar radiation, wind speed, relative humidity, cloud cover, and wind direction. The main thermophysical parameters are space coordinates, density, specific heat, conductivity, thickness, convection method, emissivity, absorptivity, and initial temperature.

### 2.2. Design of 3D Target Temperature Field Prediction Model Based on Heterogeneous Data Fusion

Predictive modelling of temperature fields based on machine learning is essentially a fitting of first-principle models of thermodynamics. According to the analysis in Section 2.1, this mainly includes three heat transfer processes: heat conduction, heat radiation, and heat convection.

In the first-principles model, the factors affecting the temperature of the model can be divided into two categories: the first category is meteorological parameters, which are time series data that record meteorological indicators at each moment, such as atmospheric temperature, wind speed, and direction, which characterizes the energy exchange between the object and the atmosphere, mainly reflects the heat radiation process and the heat convection process in the heat transfer process; the other is thermophysical parameters. If the object is regarded as composed of countless particles, then the thermophysical parameters can be regarded as a kind of point cloud data, which record the emissivity, thickness, and specific heat of each particle, which characterizes the energy exchange between points in the object and mainly reflects the heat conduction process in the heat transfer process. In addition, the spatial location distribution will cause occlusion and other phenomena and will also affect the exchange of energy. Therefore, the geometric structure information will also affect the distribution of the temperature field. Although it is not a thermophysical parameter, it corresponds to each point, so we classify it as a thermophysical parameter. These two types of data are heterogeneous and determine the temperature field distribution of the 3D model.

The existing temperature field prediction model based on machine learning only considers the influence of meteorological parameters on the temperature of the target model, while ignoring the influence of thermophysical parameters, which is equivalent to considering only the thermal convection and thermal radiation models in the first principle, while ignoring heat transfer. This results in poor prediction accuracy. We introduced a Transformer [13] to solve this problem.

The Transformer is a classic work by Google. It completely abandons the traditional neural network structure and uses an attention module [14] to process data. The use of self-attention to process data, which can effectively integrate is effective for integrating heterogeneous data.

**Figure 2:**
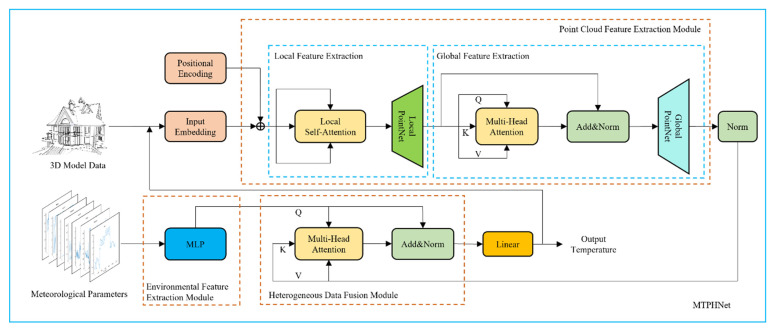
Structure of the multivariate temperature field prediction network based on heterogeneous data (MTPHNet). MLP: Multilayer perceptron.

This study comprehensively considers the thermophysical and external meteorological parameters that affect the temperature of a 3D target model. The proposed MTPHNet method improves the structure of the Transformer model using meteorological parameters as the input of the encoder, and thermophysical parameters as the input of the decoder. It uses the self-attention module to fuse the two parts of data to improve the generalization ability of the model. The structure of MTPHNet is shown in Figure 2.

The use of MTPHNet to predict the model temperature field can be expressed by Equation (5):

(5) $$Y_{temp}=\psi_{t}\left(\phi \left(Enc\left(x_{obj}\right),Dec\left(x_{env}\right)\;\right)\right)\;$$

where $\;x_{obj}$ denotes the thermophysical parameters of the 3D model, such as space coordinates, thermal conductivity, and reflectivity; $x_{env}$ denotes meteorological parameters, such as atmospheric temperature, wind speed, and direction; ϕ denotes the fusion process of thermophysical parameters and meteorological parameters to obtain fusion features; and $\psi_{t}$ represents the regression prediction process, which calculates the temperature value to be predicted.

In this study, the 3D target model is represented as point cloud data. Each data point is considered an object in space and has its corresponding attribute information, such as material, thickness, and thermal conductivity. Therefore, $x_{obj}\in {\mathbb{R}}^{P\times A}$, where *P* denotes the number of points in the 3D target model, and *A* denotes the number of attributes corresponding to each data point. Meteorological parameters are time-related, and each moment corresponds to a set of meteorological data. Therefore, $x_{env}\in {\mathbb{R}}^{T\times E}$, where *T* denotes the duration, which is obtained by sampling at a fixed step in a period, and *E* denotes the number of attributes used to describe the external environment.

It is evident that thermophysical and meteorological parameters are two sets of heterogeneous data with different dimensions. However, in the natural environment, meteorological parameters act on the thermophysical parameters of the 3D model. Simultaneously, the temperature of each data point is affected by the temperature of other points around it. Therefore, the thermophysical parameters of the 3D target model affect each other. These highly coupled heterogeneous data determine the temperature of the 3D target model; therefore, this complexity cannot be handled by general data fusion methods. Thus, we use the thermophysical parameters of each point as input to the Transformer encoder. A point corresponds to a token, and the interaction between the points of the 3D target model is simulated using the encoder’s calculation. Subsequently, the meteorological parameters are used as input to the Transformer’s decoder for feature mapping. Finally, the two parts of the features are fused, and the fused features are regressed to calculate the predicted value of the 3D target temperature field.

#### 2.2.1. Point-Cloud Feature Extraction Module (PCEM)

In the real environment, objects can be envisioned as a composition of countless particles, and different points have different materials and spatial positions. Different materials will often have very distinct emissivity, absorption, and scattering [15] properties, which can result in a variety of particle energy absorptions and releases. Different spatial positions will lead to phenomena, such as occlusion and shadows, resulting in uneven energy distributions. Therefore, the thermophysical parameters of the 3D object are crucial to the establishment of a temperature field. To improve the accuracy of temperature field prediction, we must extract the object’s thermophysical parameters.

The thermophysical parameters include spatial coordinates, emissivity, and specific heat, which can be regarded as point cloud data with additional attributes. Temperature field prediction requires the calculation of the entire 3D target model, and each point interacts with all other points, implying that the thermophysical parameters of each are dot-produced with the thermophysical parameters of other points. The computational complexity of the original Transformer is proportional to the square of the length of the input sequence [16]; however, the number of 3D point cloud points is large, which is unsuitable for most hardware.

To solve this problem, we apply PointNet [17], a feature extraction layer for point-cloud data of the 3D target model. PointNet, proposed by Qi et al. (2017), can be directly used to process point cloud data. The model extracts features via feature mapping and maximum pooling of point cloud data and satisfactorily completes the classification task. However, because the model extracts features from single points and does not consider the relationship between points, its local feature extraction ability is weak [18]. Therefore, it is impossible to analyze complex scenes.

**Figure 3:**
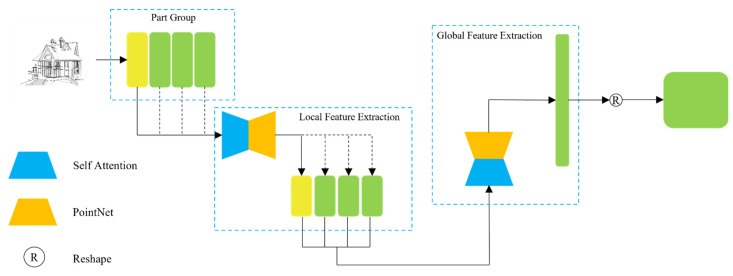
Flow chart of feature extraction of 3D target model.

In this study, the 3D target model is assembled from different parts. First, we group the point clouds of the 3D target model according to the types of parts. The point cloud attributes of the same part are similar; however, the point cloud attributes of different parts are different. Subsequently, the point cloud data are organized according to the part category and each group of point cloud data are first sent to a self-attention module for calculation to obtain the relationship between points. The calculation results are sent to the PointNet for local feature extraction. The feature extraction process for the point cloud data of the 3D target model is shown in Figure 3.

Figure 3 indicates that we do not configure a self-attention module and PointNet, respectively, for the point cloud data of each group of parts to extract features. We rather refer to the convolution kernel of weight sharing in convolutional neural network [19] and use a unique self-attention module and PointNet which perform feature extraction on the point cloud data of different parts. Each group of parts is extracted as a local feature vector; the feature items extracted from all the parts are formed into a new sequence; and are then sent to a new self-attention module and PointNet to extract global features. This way, all features for the 3D target model are extracted.

#### 2.2.2. Environmental Data Feature Mapping Module (EMM)

Meteorological parameters directly affect the temperature of objects. Rain reduces the surface temperature of objects, the shielding effect of clouds weakens solar radiation, and wind accelerates the heat transfer between the air and the surface of the object [20].

**Figure 4:**
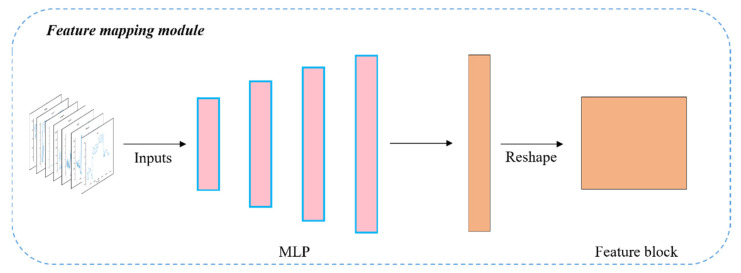
Schematic of environmental data features mapping module.

The thermophysical parameters of the 3D target model are mapped into a fixed-size feature block after passing through the PCEM. The thermophysical and meteorological parameters of the 3D model are heterogeneous data from different sources. To achieve the integration of heterogeneous data, we introduced a multi-layer perceptron (MLP) module to map meteorological parameters to a high-dimensional space through feature mapping and map them to a fixed size to match the feature block of the thermophysical parameters. The EMM is illustrated in Figure 4.

#### 2.2.3. Data Fusion Module (DFM)

**Figure 5:**
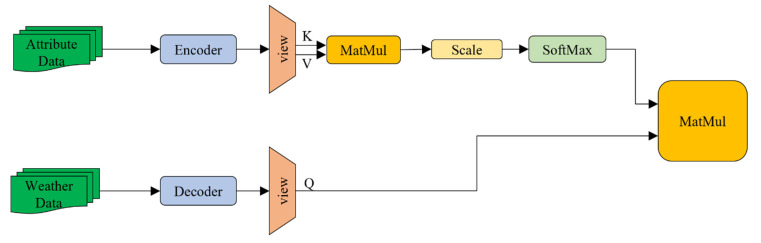
Schematic of the integration of internal parameter variables and external environmental parameter variables.

In the natural environment, meteorological and thermophysical parameters undergo complex physical interactions to determine the temperature field distribution of objects. In this study, we use a self-attention module to fuse the thermophysical and meteorological parameters. We use the feature block output by the encoder as the K and V of the self-attention module, and the feature block output by the decoder as the Q of the self-attention module. This process simulates the interaction between meteorological parameters and the 3D target model in the natural environment. A schematic of the integration of thermophysical and meteorological parameters is shown in Figure 5.

#### 2.2.4. Pseudocode

Based on the above analysis, the pseudocode of MTPHNet shown in Figure 2 is summarized, and the algorithm is given in Algorithm 1.

**Algorithm 1** program pseudo code of MTPHNet.
**Input**
$x_{env}^{t}$: meteorological parameter at the current moment. $x_{obj}^{t}$: thermophysical parameter at the current moment. $Y_{temp}^{t-1}$: the target temperature value at the last moment.
**Output**
$Y_{temp}^{t}$: the target temperature value at the current moment.
1 **For**
t=1 to $t_{max}$
2   Replace: $x_{enc\underline{}in}^{t}=dimension\_replace\left(x_{obj}^{t},Y_{temp}^{t-1}\right)$
3   **For** i=1 to P
4     $x_{enc\underline{}in}^{t}\left[i\right]=attn\_feature\left(x_{enc\underline{}in}^{t}\left[i\right],x_{enc\underline{}in}^{t}\left[i\right],x_{enc\underline{}in}^{t}\left[i\right]\right)$
5     $x_{enc\underline{}in}^{t}\left[i\right]=pointnet\_feature\left(x_{enc\underline{}in}^{t}\left[i\right]\right)$
6   **End for**
7   $x_{enc\underline{}out}^{t}=\;pointnet\_feature\left(attn\_feature\left(x_{enc\underline{}in}^{t},x_{enc\underline{}in}^{t},x_{enc\underline{}in}^{t}\right)\right)$
8   $x_{dec\underline{\;}in}^{t}=MLP\left(x_{env}^{t}\right)$
9   $Y_{temp}^{t}=\;Linear\left(attn\_feature\left(x_{dec\underline{}in}^{t},x_{enc\underline{}out}^{t},x_{enc\underline{}out}^{t}\right)\right)$
10 **End for**

Algorithm 1 shows that $t_{max}$ is the maximum duration of temperature field prediction, and P is the number of parts in the 3D model.

In line 2, the algorithm replaces the dimension representing temperature in $x_{obj}^{t}$ with $Y_{temp}^{t-1}$. From lines 3 to 6, the algorithm extracts the local features of the 3D model using attn_feature and pointnet_feature for each part. In line 7, the algorithm uses attn_feature and pointnet_feature to extract the global features of the 3D model. In line 8, the algorithm uses MLP to make a feature map of $x_{env}^{t}$. Finally, the algorithm uses attn_feature to fuse $x_{dec\_in}^{t}$ and $x_{enc\_out}^{t}$, and Linear to obtain the temperature value.

## 3. Experimental Details and Data Exploitation

### 3.1. Experimental Environment and Index Design

The experiment was conducted on an AMD Ryzen 7 CPU 5800H with 16 GB of RAM, NVIDIA GeForce RTX 3090 with 24 GB of memory, Python 3.7.2, and PyTorch 1.9.0 for network model training and testing.

To evaluate the effect of temperature field prediction, mean absolute error (MAE) [21], root mean square error (RMSE) [22], and $R^{2}$ [23] were selected as the evaluation criteria for the model quality. The calculation formulas are as follows:

(6) $$MAE\left(y,\hat{y}\right)=\frac{1}{n}\overset{n}{\underset{i=1}{\sum }}\left|y_{i}-\hat{y_{i}}\right|$$

(7) $$RMSE\left(y,\hat{y}\right)=\frac{1}{n}\overset{n}{\underset{i=1}{\sum }}\|y_{i}-\hat{y_{i}}{\|}_{2}^{2}$$

(8) $$R^{2}\left(y,\hat{y}\right)=1-\frac{RSS}{TSS}=\frac{ESS+2\sum_{i=0}^{n}\left(y_{i}-\hat{y_{i}}\right)\left(\hat{y_{i}}-\bar{y}\right)}{TSS}$$

(9) $$TSS=\overset{n}{\underset{i=1}{\sum }}{\left(y_{i}-\bar{y}\right)}^{2}$$

(10) $$RSS=\overset{n}{\underset{i=1}{\sum }}{\left(y_{i}-\hat{y_{i}}\right)}^{2}$$

(11) $$ESS=\overset{n}{\underset{i=1}{\sum }}{\left(\hat{y_{i}}-\bar{y}\right)}^{2}$$

where y denotes the true value; $\hat{y}$ denotes the predicted value; $\bar{y}$ denotes the average value of the true value; TSS is the Total sum of squares, which defines the difference between y and $\bar{y}$; RSS is the Residual sum of squares, which defines the difference between y and $\hat{y}$; and ESS is the Explained sum of squares, which defines the difference between $\hat{y}$ and $\bar{y}$.

Among the selected evaluation indicators, MAE and RMSE are used to measure the size of error between the predicted and real data; R-squared measures the quality of the fit.

### 3.2. Dataset

The training data used by existing temperature field prediction models based on machine learning methods were collected by instruments. These type of data are closer to reality. However, owing to the variability of natural environmental parameters and the in-stability of instruments, the data acquired by the instrument are noisy and costly.

We use our own temperature dataset constructed by ourselves, which includes the thermophysical parameters, meteorological parameters, and temperature field data of 3D objects.

#### 3.2.1. Dataset Format

We use the thermophysical and meteorological parameters of the dataset as input to MTPHNet and the corresponding temperature data as its output to train and optimize the model parameters.

**Figure 6:**
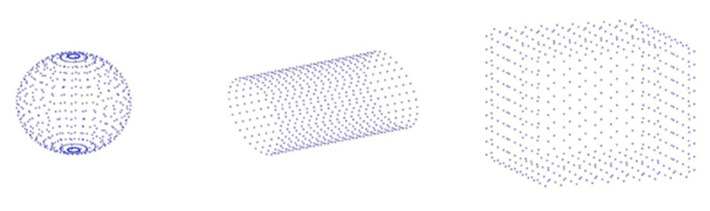
A 3D model and its corresponding point cloud data.

The shape of the 3D target model has an impact on the temperature field formation. Under the same environmental conditions, different shapes will cause uneven heat distribution in the 3D target model, for instance, objects in shadow will be cooler than objects in direct sunlight. Therefore, the thermophysical parameters in the dataset first need to obtain the spatial position information of the 3D target model. We built several 3D models using 3D modeling software and exported them to OBJ file format. Because OBJ file uses the face element data structure to build the 3D model and the proposed model uses the point cloud data structure, we processed the exported OBJ file and calculated the center coordinates of each face element to replace the face element. Figure 6 shows the constructed 3D model.

Figure 6 shows that each 3D object has several data points. In addition to spatial coordinates, each data point contains additional attribute information, such as material, thickness, and initial temperature. Table 1 shows the point cloud data format of the 3D target model during training.

**Figure 7:**
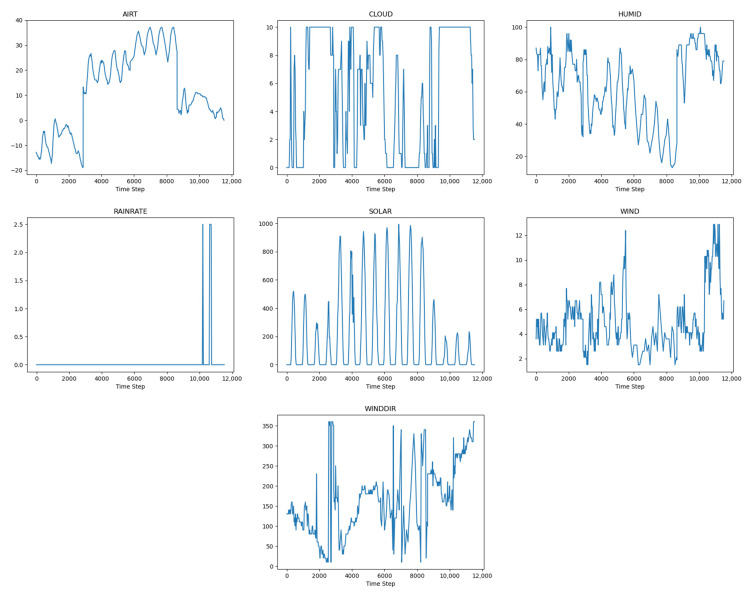
External meteorological parameters for temperature field prediction. Parameters from the left to right and top to bottom are atmospheric temperature, cloud cover, relative humidity, rainfall rate, solar radiation, wind speed, and direction.

In addition to the 3D point cloud data, meteorological parameter data are required. For this study, we collect meteorological parameter data for four seasons. Combined with the parameters that must be collected in the analysis above, we selected date, atmospheric temperature, solar radiation, wind speed and direction, relative humidity, and cloud coverage as environmental parameter variables. Figure 7 shows the meteorological parameters related to time, and the changing trends.

According to the collected thermophysical and meteorological parameters, we use an internal temperature field calculation software to calculate the temperature field distribution of the 3D model data and add the calculation results to the dataset for training the model.

From Equation (5), $Y_{temp}\in {\mathbb{R}}^{P\times T}$, which means *P* points, and each point has *T* temperature values.

#### 3.2.2. Teacher Forcing

As discussed before, the temperature of the 3D target is affected by the sun, atmosphere, and surrounding objects. It is evident that the temperature of the 3D target model at each moment is determined by the meteorological and thermophysical parameters at the current moment.

Among the features of the thermophysical parameters, one dimension of the feature represents the temperature of the point cloud data. Because the 3D model is represented by a point cloud, each point represents a distinct object. Therefore, this dimension represents the distribution of the temperature field at the current moment.

The temperature field distribution is obtained at the next moment by entering the data into MTPHNet to measure the difference between the two one time step.

**Figure 8:**
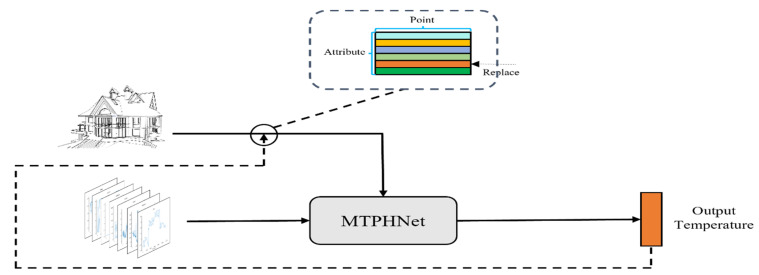
Temperature substitution process. The temperature value calculated by the model replaces a certain dimension of the input to simulate the temperature change of all objects in the temperature field at each moment.

Because unknown information cannot be used in the test, the calculated value is assigned to this dimension of the input data to calculate the temperature value at the next moment, after calculating the temperature value at the current moment. Figure 8 illustrates the process.

During the training, the temperature value at any time is known. Therefore, there is no need to use the temperature value calculated by the model to replace the value of the dimension, which allows parallel calculations during the training.

## 4. Results and Discussion

### 4.1. Performance of the MTPHNet

To demonstrate that the MTPHNet successfully integrates an object’s thermophysical and meteorological parameters can further improve the prediction of the temperature field, we used temperature field data, thermophysical parameters, and meteorological parameters as training data and compared the performance of MTPHNet with those of v-SVR and a combined BP neural network (CBPNN) model.

**Figure 9:**
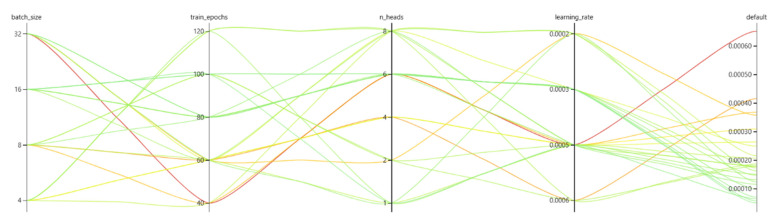
Results of NNI.

When training MPTHNet, the hyperparameters needed by the model included batch size, epoch, number of multi-heads, and initial learning rate. The batch size affects the degree of optimization and model speed. The size of the epoch affects the fitting effect of the model. Tuning the number of multi-heads helps the network capture richer features. The initial learning rate determines if and when the objective function converges to a local minimum. To obtain better hyperparameter values, we used Microsoft’s automatic parameter tuning tool, NNI, for hyperparameter selection, which runs the code in a loop to obtain the optimal hyperparameter values. The results of the operation are shown in Figure 9:

As can be seen in Figure 9, the batch size was set to 16; the number of epochs was 100, the number of multi-heads was 6 and the initial learning rate was 0.0003. As it is based on the Transformer structure, the MTPHNet model is large and needs a significant amount of memory. Considering computational efficiency and fitting accuracy, we selected the Huber loss function and the Adam optimizer for optimization. Dropout was used for overfitting mitigation, and the deletion ratio, *p*, was 0.05.

Because the thermophysical parameters of the 3D target were considered, the MTPHNet trained different 3D models with the same number of point clouds. However, v-SVR and CBPNN only consider the impact of meteorological parameters on the temperature of the 3D target and cannot simultaneously predict the temperature field of different 3D target models. For referential significance, all three models were trained with the same training set, which includes the temperature field distribution data of a single 3D model. MTPHNet was better than the other prediction models after testing on the test set. Table 2 presents the generalization performance of the models.

**Figure 10:**
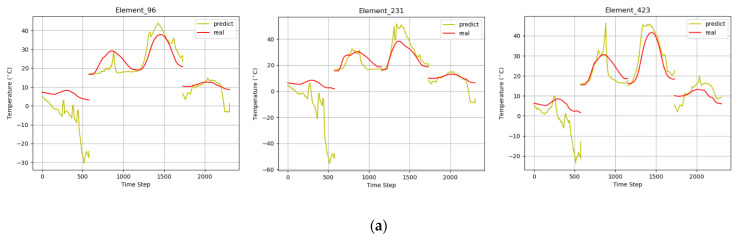

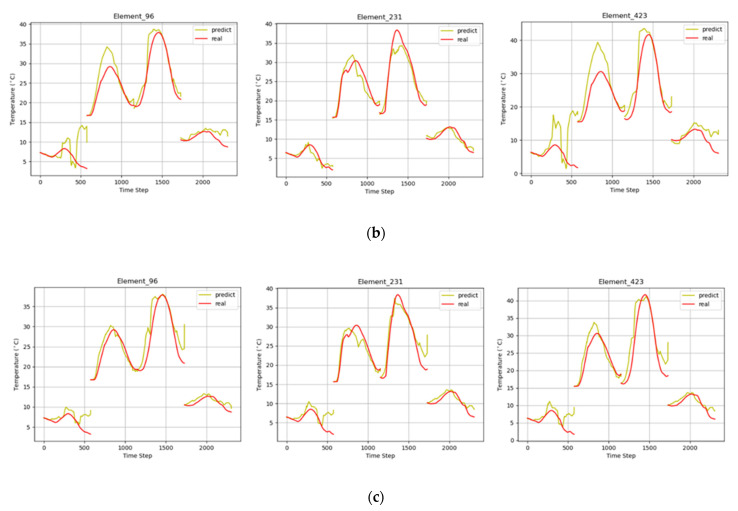
Generalization performance renderings. Prediction effect diagrams of (**a**) v-SVR; (**b**) CBPNN; and (**c**) MTPHNet.

As shown in Table 2, the MTPHNet prediction error, was significantly lower than that of the existing temperature field prediction models. Compared with the CBPNN model, its MAE and RMSE decreased by 23.4% and 27.7%, respectively, whereas the R-squared increased by 5.85%. Figure 10 shows the prediction effects of the models.

Because the 3D model was composed of patches, in the experiment, we extracted several patches by generating random numbers to show the effect of temperature field prediction. We selected patches 96,231 and 423 for presentation. The experiments demonstrated that, although the existing temperature field prediction methods fit the temperature field on the change trend, their accuracies were insufficient. Therefore, it is necessary to combine the energy interaction mode of the 3D object in the natural environment and its meteorological and thermophysical parameters to further improve prediction accuracy.

### 4.2. Advantages of MTPHNet

#### 4.2.1. Multi-Object Temperature Field Prediction

The results indicate that MTPHNet has a better fitting ability than the existing temperature field prediction models. Because MTPHNet comprehensively considers the various energy exchanges between the object and the environment and combines the thermophysical parameters of the object for training, it simultaneously trains and predicts the temperature field for different 3D targets. Existing temperature field prediction models cannot achieve this.

In this study, we summarized the 3D target temperature field data shown in Figure 5 and imported them into MTPHNet for training and verification. Table 3 presents the fitting performances.

**Figure 11:**
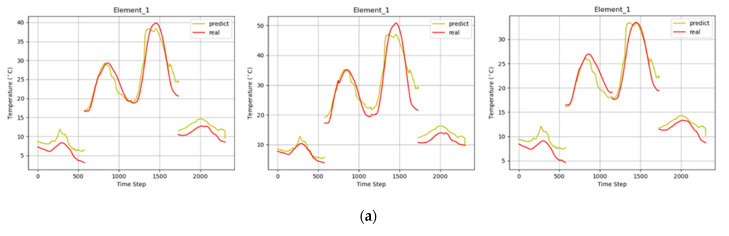

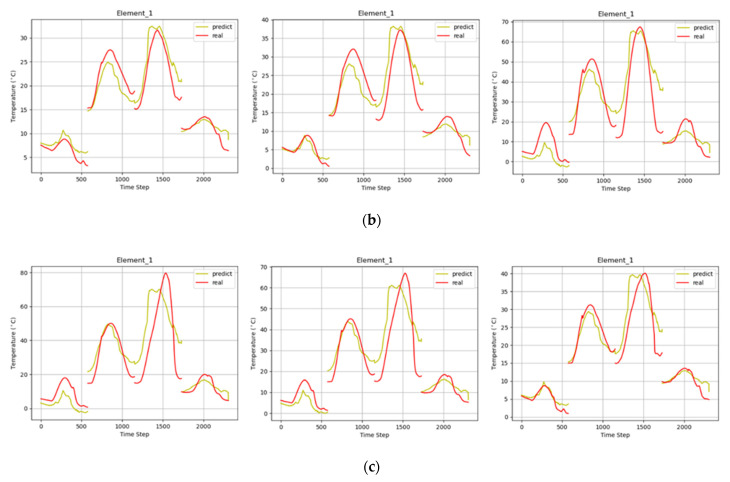
Multi-object temperature field prediction effects. Fitting of different materials of (**a**) box; (**b**) cylinder; and (**c**) sphere.

As shown in Table 3, when the MTPHNet model was used to predict the temperature field of multiple objects, the values of its various indicators were satisfactory. The experimental results demonstrate that the thermophysical parameters of the 3D target model are significant for temperature field prediction. Figure 11 shows the effects of the multi-object temperature field prediction. Here, three materials were used for the temperature field calculation. For a convenient comparison, we selected patch 1 for presentation. The same patch shows the effect of different materials on temperature and the adaptability of MTPHNet to different temperature changes.

#### 4.2.2. Prediction of Temperature Field of Complex Objects

**Figure 12:**
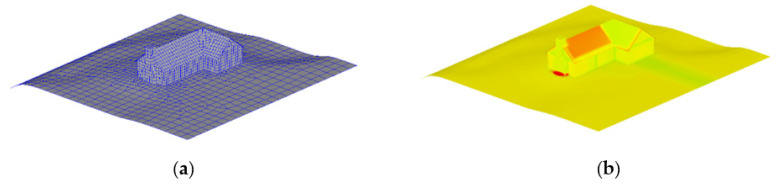
Complex house model with 5660 patches and 30 parts: (**a**) geometric structure; (**b**) temperature field distribution at a given moment.

When predicting multiple objects, this study assumed that each object had only one part; thus, the attribute data of different points are the same. In reality, however, a complex 3D object is composed of different materials, and the energy exchange between them is more complicated than that of a single material. Therefore, we chose a complex model for training and prediction. Figure 12 shows the geometry of the model.

**Figure 13:**
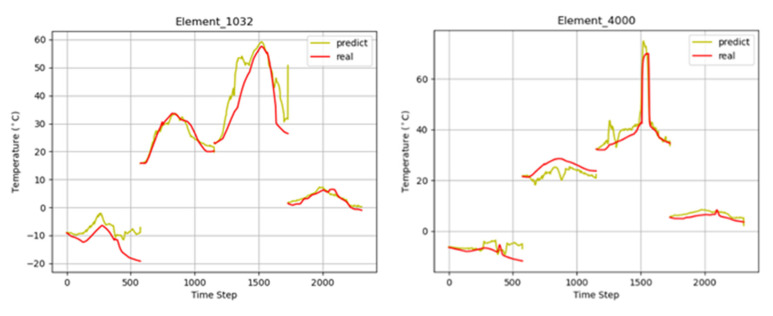
Prediction of the temperature field of complex objects.

Table 4 demonstrate that the model has a good generalization performance for the temperature field prediction of complex models, which further reflects the superiority of MTPHNet. Figure 13 shows the prediction effect of the temperature field of complex objects. We randomly selected patches 1032 and 4000 for presentation.

### 4.3. Ablation Analysis

**Figure 14:**
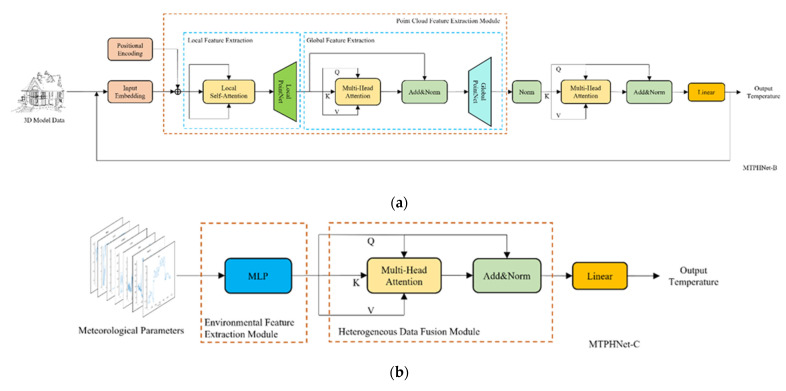

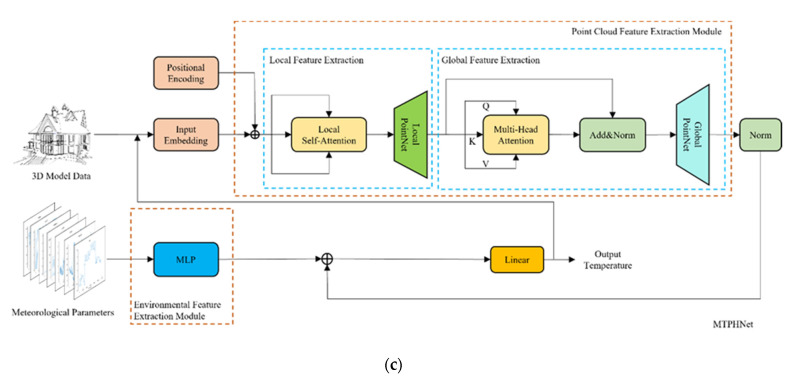
Multivariate temperature field prediction network based on heterogeneous data (MTPHNet) variants for ablation experiments: (**a**) MTPHNet-B removes the environmental data feature mapping module (EMM) to study the effect of meteorological parameters on temperature field prediction; (**b**) MTPHNet-C removes the point cloud feature extraction module (PCEM) to study the effect of thermophysical parameters on temperature field prediction; and (**c**) MTPHNet-D re-places the data fusion module (DFM) with an additive fusion method to study the effect of data fusion on temperature field prediction.

To verify the effectiveness of our proposed network, we conducted three ablation experiments to verify the performance of the main design components: environmental data feature mapping module (EMM), point cloud feature extraction module (PCEM), and data fusion module (DFM). The proposed MTPHNet is given as MTPHNet-A, and its variants for ablation are MTPHNet-B, MTPHNet-C, and MTPHNet-D. All variants were trained and validated using the same procedure described in Section 4.1. Each ablation experiment was performed three times and the results were averaged, and shown in the Table 5 and Figure 14.

#### 4.3.1. Effectiveness Analysis of EMM

To measure the EMM’s contribution, we designed a variant model without EMM, as described in Table 5: MTPHNet-B. It can be seen that the prediction effect of MPTHNet-A without EMM is extremely poor; it cannot even predict the temperature. The quantitative results show that the EMM is the core of temperature prediction.

#### 4.3.2. Effectiveness Analysis of PCEM

We believe the use of PCEM would further improve the accuracy of temperature prediction. To substantiate it, we designed a variant without the PCEM: MTPHNet-C. In Table 5, MTPHNet-A outperforms MTPHNet-C in all metrics. The quantitative results clearly show that PCEM improved the prediction performance.

#### 4.3.3. Effectiveness Analysis of DFM

DFM fuses the features extracted from meteorological and thermophysical parameters, which is a crucial step. To confirm this, we designed a variant model, MTPHNet-D, which replaces the DFM with an additive fusion module. In Table 5, MTPHNet-A outperforms MTPHNet-D in all metrics, and MTPHNet-D is closer to MTPHNet-C in terms of metrics. The quantitative results show that DFM and PCEM contribute similarly to improve the prediction performance.

## 5. Conclusions

This study comprehensively considered the thermophysical and meteorological parameters affecting the temperature field distribution of a 3D target. Combined with temperature field distribution data, an intelligent temperature field prediction model, MTPHNet, was proposed. To fuse meteorological and thermophysical parameters, MTPHNet used PCEM to calculate the interaction between 3D target attributes and extract thermophysical features. Simultaneously, it used EMM to map meteorological parameters to meteorological features so that the mapped data and thermophysical data would be of the same size, which facilitated the subsequent data fusion. Finally, DFM fused the parts and used the results to predict the temperature. Considering PCEM’s tendency of memory explosion when processing point cloud attribute data, we introduced PointNet as a feature extraction network to reduce the memory burden and divide the feature extraction process into local feature and global feature extraction activities to further streamline memory use. Compared with v-SVR and CBPNN, the MAE and RMSE of MTPHNet were reduced by at least 23.4% and 27.7%, respectively, whereas the R2 value increased by at least 5.85%. The results show that MTPHNet effectively improves model generalizability to more efficiently and accurately predict temperature fields while meeting real-time infrared simulation processing requirements. In complex object temperature field prediction tasks that simulate real environments, MTPHNet is advantageous in that it considers realistic energy interaction processes. Its MAE, RMSE, and R2 values were 2.645, 3.522, and 0.964, respectively, demonstrating the model’s high adaptability to real scenes.

It should be noted that when MTPHNet performs multi-model prediction tasks, the number of point clouds of different 3D models are required to be the same, which significantly increases the difficulty of data collection. Therefore, in a future work, we plan to change the model structure so that it can be further adapted to 3D models varying numbers of point clouds.

## Figures and Tables

**Table 1:** Point cloud data format for 3D targets.

Physical Parameters	Space Coordinates	Density	Specific Heat	Conductivity	Thickness	Convection	Emissivity	Absorptivity	Initial Temperature
Unit	(mm)	(kg/m^3^)	(J/kg·K)	(W/m·K)	(mm)	Bool	/	/	°C

**Table 2:** Comparison of generalization performance of MTPHNet, v-SVR, and CBPNN.

Algorithm Model	MAE	RMSE	R-Squared
v-SVR	17.329	21.17	−388.6
CBPNN	2.249	3.474	0.889
MTPHNet	1.722	2.512	0.941

**Table 3:** MTPHNet’s generalization performance for multi-object temperature field prediction.

Model	Material	MAE	RMSE	R-Square
Box	1	2.077	2.568	0.938
	2	3.953	5.664	0.877
	3	1.785	2.153	0.929
Cylinder	1	4.419	6.224	0.855
	2	2.497	3.320	0.901
	3	5.572	7.976	0.821
Sphere	1	1.910	2.329	0.918
	2	2.556	3.245	0.897
	3	4.843	6.927	0.831

**Table 4:** MTPHNet’s generalization performance for temperature field prediction of complex objects.

Model	MAE	RMSE	R-Square
House	2.645	3.522	0.964

**Table 5:** Quantitative evaluation metrics of MTPHNet and its variants. All models follow the same procedure and training environment as described in Section 4.1 and are evaluated on the same test set. The best results are shown in bold.

Model	MAE	RMSE	R-Square
**MTPHNet-A (Original)**	**1.722**	**2.512**	**0.941**
MTPHNet-B (no EMM)	8.734	10.362	−0.011
MTPHNet-C (no PCEM)	2.277	3.516	0.885
MTPHNet-D (no DFM)	2.303	3.431	0.89

## Data Availability

The data used to support the findings of this study are available from the corresponding author upon request.
